# THE EXPERIENCE OF SOCIAL WORKERS IN BIG DATA–BASED CARE SERVICES ON PREVENTING SOCIAL ISOLATION FOR OLDER ADULTS

**DOI:** 10.1093/geroni/igae098.2992

**Published:** 2024-12-31

**Authors:** Haklyoung Kim, Boram Hwang, Sungwan Kang, Seongsu Choi, Kyoungmi Mun, Yeong Ju Lee

**Affiliations:** Pusan National University, Busan, Republic of Korea; Pusan National University, Busan, Republic of Korea; Pusan National University, Busan, Republic of Korea; Pusan National University, Busan, Republic of Korea; Pusan National University, Busan, Republic of Korea; Pusan National University, Busan, Republic of Korea

## Abstract

In response to the increasing occurrence of social isolation among older adults in South Korea, local governments and ICT companies are collaborating to introduce various intelligent care initiatives based on big data, including AI care calls, AI speakers, and activity detection sensors. This study investigates the experiences of social workers providing on-site intelligent care services to older individuals living alone to prevent social isolation. Five focus group interviews involving 15 welfare officials and social workers were conducted. The findings are as follows. Firstly, they recognized biases regarding intelligent technology, such as concerns about privacy infringement, as barriers to identifying high-risk groups and accepting services. Secondly, challenging living environments with limited internet access and low digital literacy were acknowledged as obstacles hindering the sustained use of intelligent care services. Thirdly, the presence of personnel capable of providing education and support for smart device usage was deemed essential for the continued utilization of services. Fourthly, it was acknowledged that additional personnel and support beyond smart devices are necessary for groups with impaired vision, hearing, or mobility. Lastly, the involvement of social workers with a deep understanding of high-risk groups, from planning to evaluation of service models, was recognized as essential for enhancing the effectiveness and efficiency of services. Based on these findings, this study suggests practical and policy implications regarding intelligent living environments, digital literacy, ethics of artificial intelligence, caregiver training, and the involvement of social workers.

